# Supplementary material for: Genomic disparities between cancers in adolescent and young adults and in older adults
Source: Nat Commun. 2022 Nov 24;13:7223. doi: 10.1038/s41467-022-34959-2 (PMC9700745; doi:10.1038/s41467-022-34959-2)
Supplement: Supplementary file 1 — Supplementary Information [file 41467_2022_34959_MOESM1_ESM.pdf]

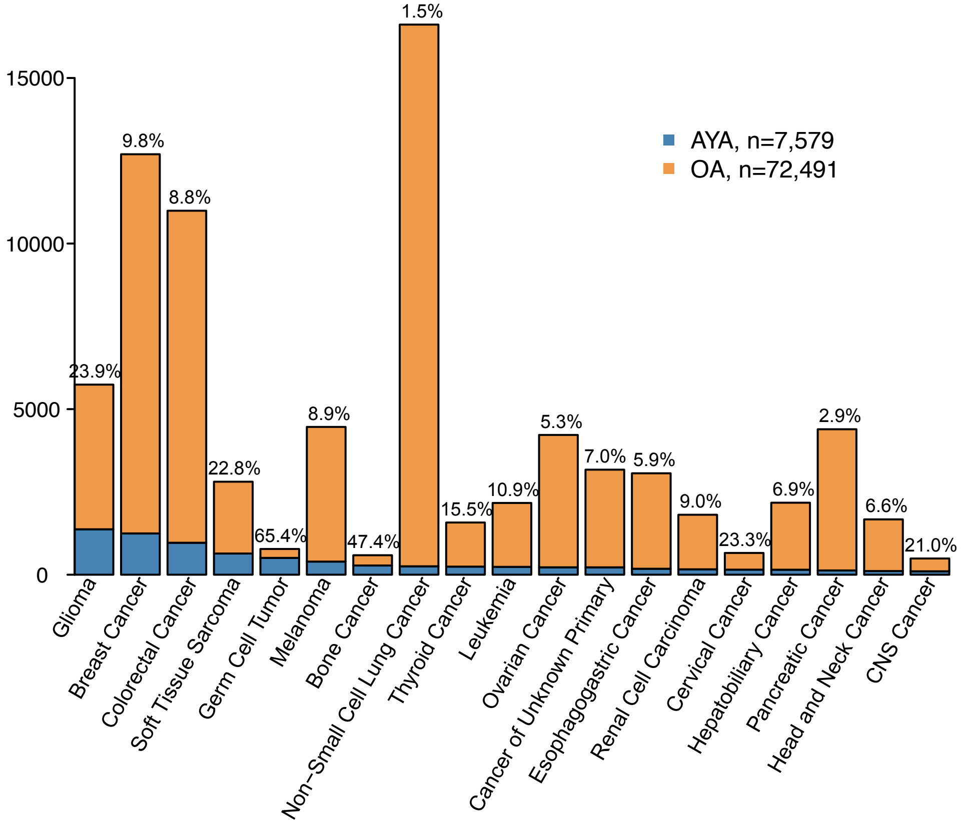


**Supplementary Fig. 1 Dataset overview.** AYA/OA sample counts by tumor type, shown in decreasing order by AYA sample count. The percentage on top of each bar indicates the percentage of AYAs in the cancer cohort.

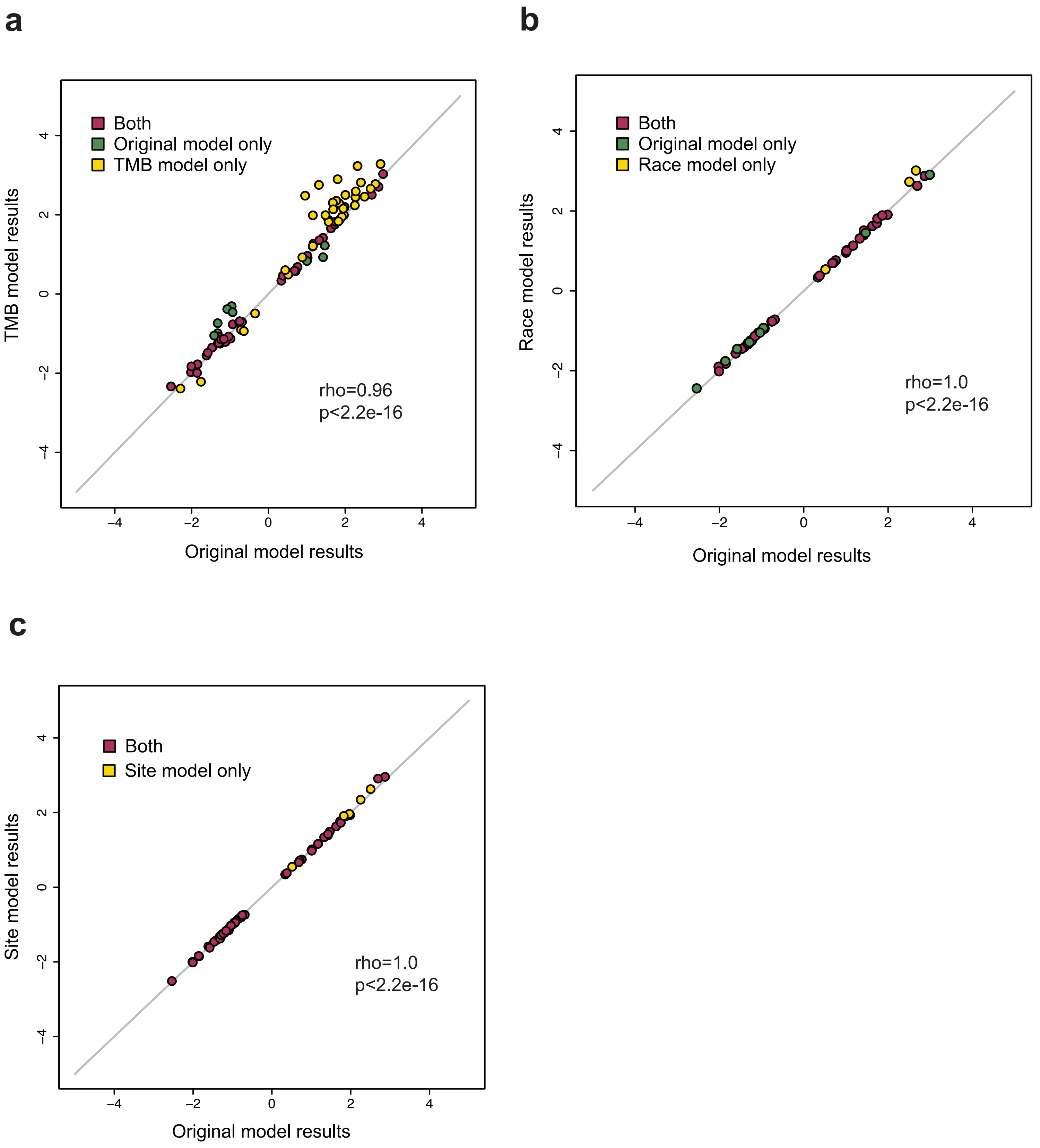


**Supplementary Fig. 2 Impact of confounding factors on AYA effect size. a.** Spearman correlation of AYA effect size between the original model (x axis) and the TMB model (y axis). **b.** Spearman correlation of AYA effect size between the original model (x axis) and the model with race/ethnicity variable (y axis). **c.** Spearman correlation of AYA effect size between the original model (x axis) and the model with added "site" variable (y axis). Each dot represents one gene-cancer pair identified from either model.

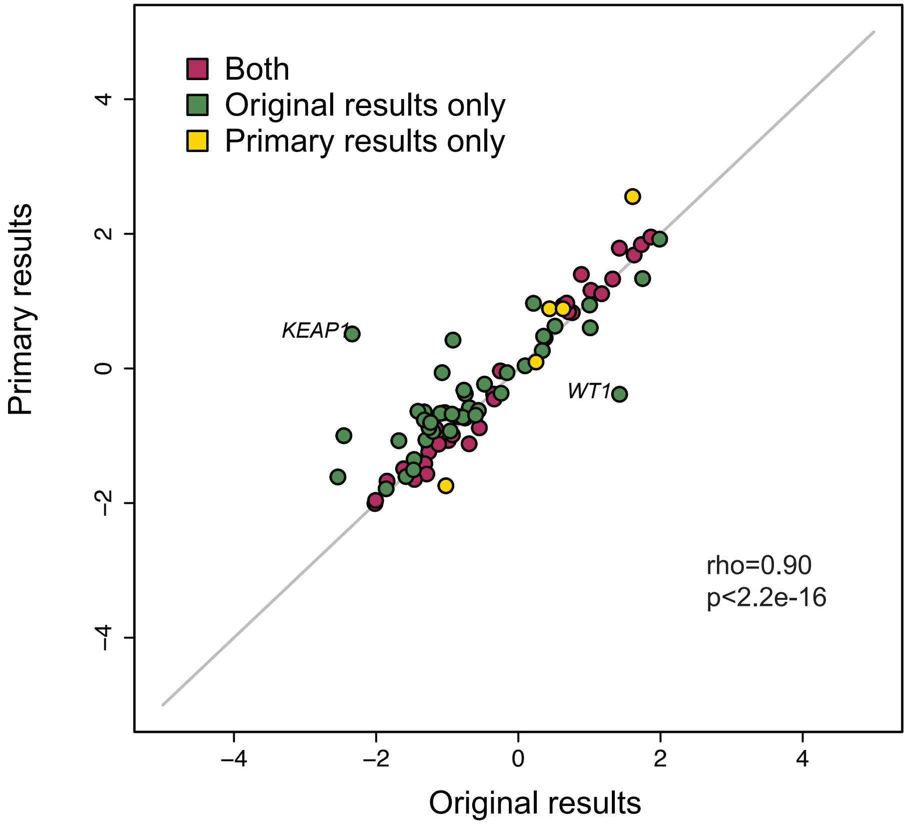


**Supplementary Fig. 3 Impact of metastasis on AYA effect size.** Spearman correlation of AYA effect size in results with and without metastatic samples. The x axis represents effect size from the original model, and the y axis represents the effect size from a model based on only primary tumors. Each dot represents one gene-cancer pair identified from all samples and primary samples only.

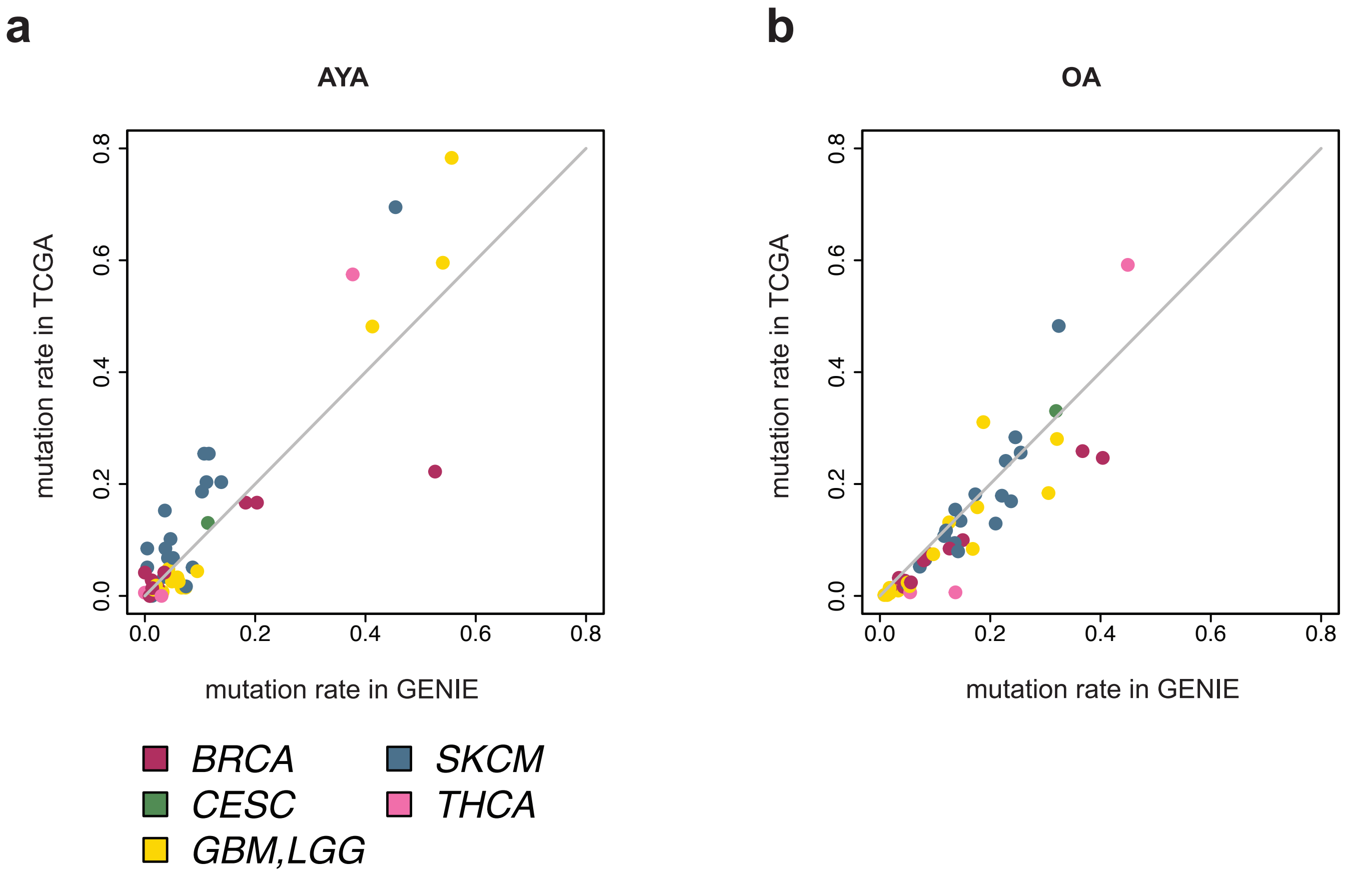


**Supplementary Fig. 4 Gene mutation rate in TCGA and GENIE. a.** Gene mutation rate in AYAs in GENIE (x axis) and TCGA (y axis). **b.** Gene mutation rate in OAs in GENIE (x axis) and TCGA (y axis). Each dot represents one gene-cancer pair that was identified showing different mutational rate between AYA and OA in GENIE dataset.

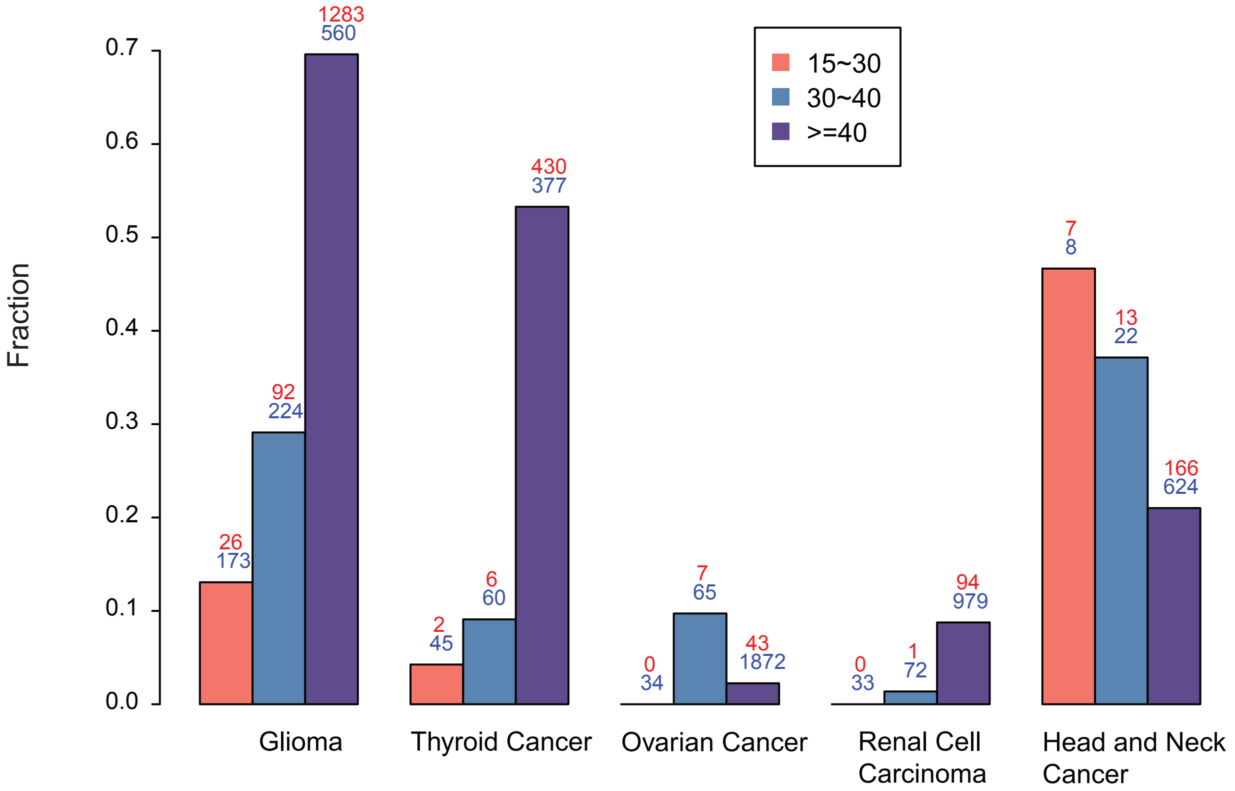


**Supplementary Fig. 5 TERTp mutation rates across age groups.** TERTp mutational frequencies of split AYA groups in five cancer types. The numbers on top of each bar represent the number of TERTp mutant (red) and wildtype samples (blue).

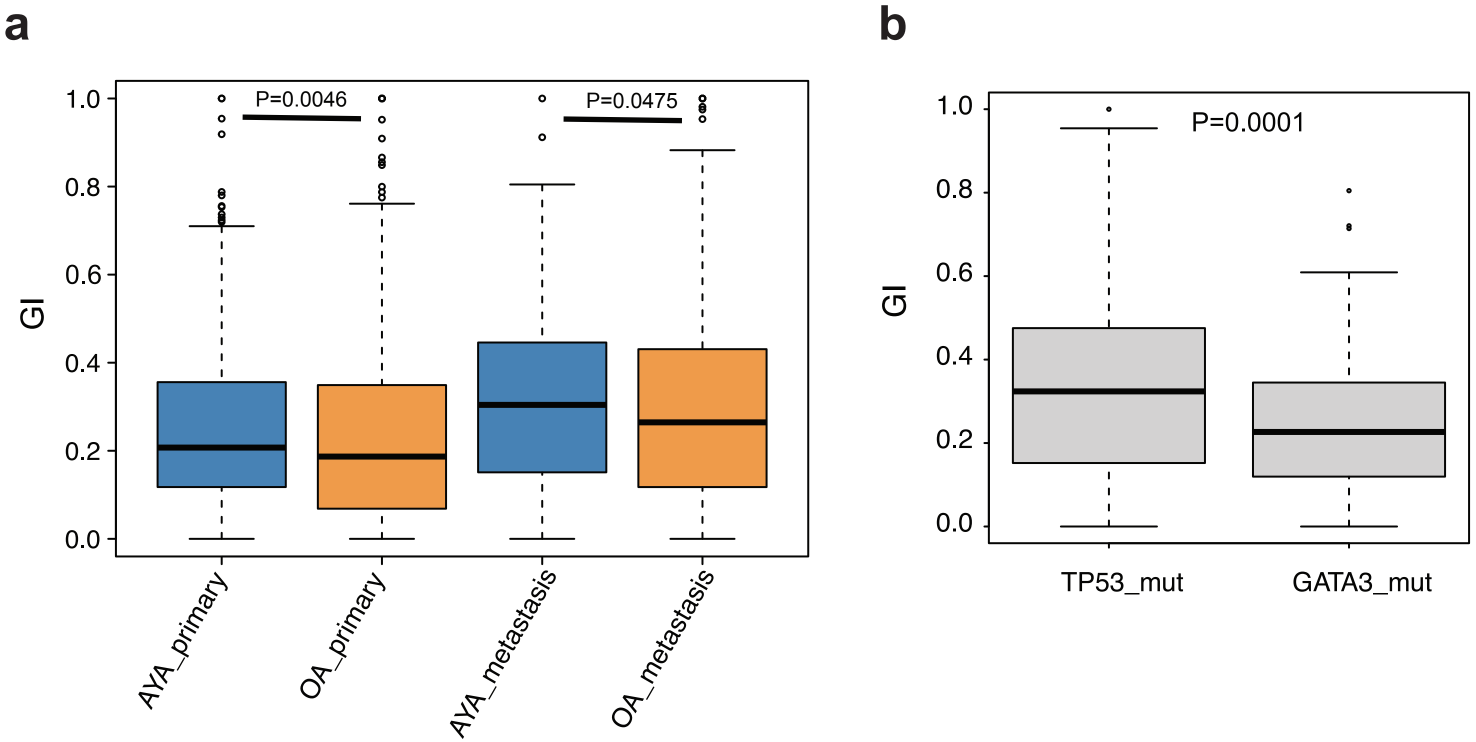


**Supplementary Fig. 6 Genomic instability in breast cancer. a.** GI score comparison between AYAs and OAs in breast cancer (left, primary tumor; right, metastasis tumor. AYA primary, n=371; OA primary, n= 2650; AYA metastasis, n=256; OA metastasis, n=2632). **b.** GI score comparison between TP53 and GATA3 mutated tumors in AYA breast cancer (TP53 mut, n=643; GATA3 mut, n=184). Boxplot center represents the median value; box limits represent upper and lower quartile; whiskers indicate the 1.5 interquartile range and points indicate outliers. Statistical differences were assessed using two-sided Wilcoxon rank sum test.

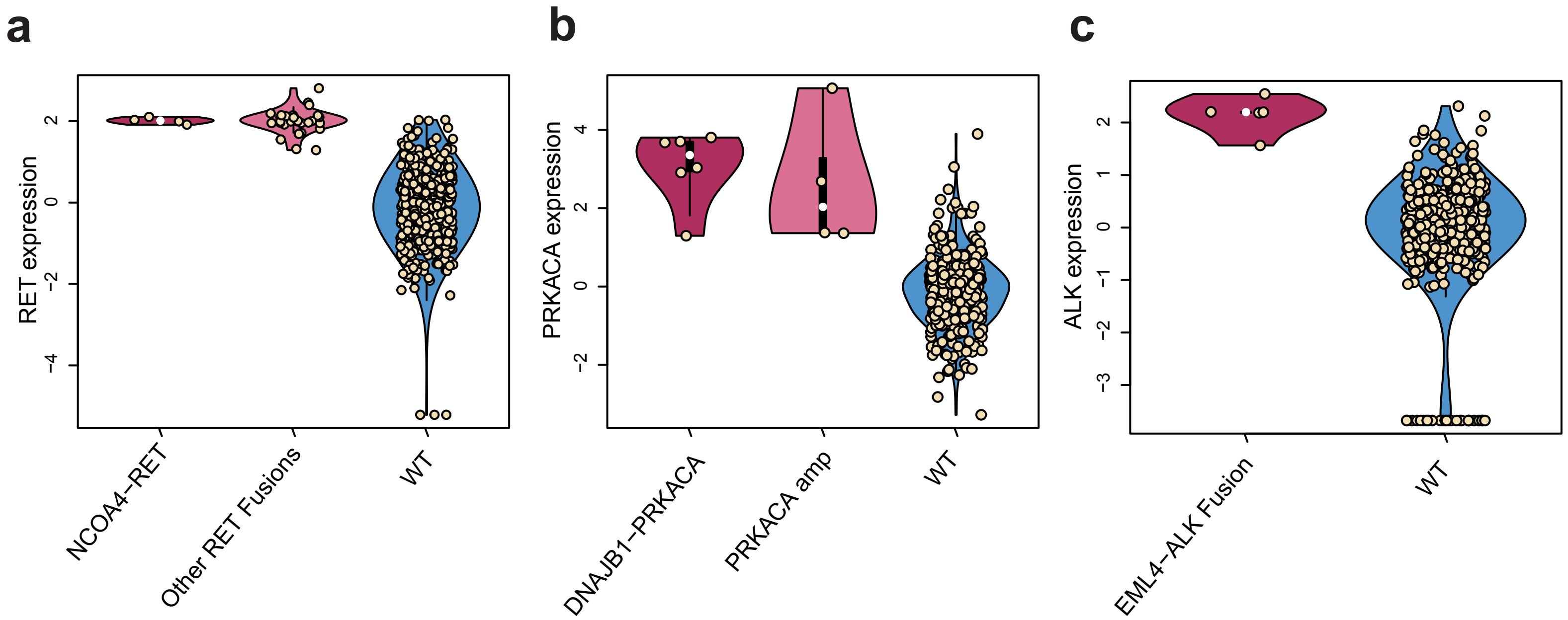


**Supplementary Fig. 7 Functional consequences of gene fusions.** The y axis represents expression level of the oncogene that is involved in the fusion. We also include tumors with other alterations of the oncogene for comparison. **a.** RET expression in THCA (NCOA4-RET fusion, n=4; other RET fusion, n=31; wt, n=463). **b.** PRKACA expression in LIHC (DNAJB1-PRKACA fusion, n=6; PRKACA amplification, n=4; wt, n=359). **c.** ALK expression in LUAD (EML4-ALK fusion, n=5; wt, n=507). Center white dot represents the median, the thick black bar in the center represents the upper and lower quartiles, whiskers represent the 1.5 interquartile range. Width of the violin represents density of data points.
