# Supplementary material for: Genomic disparities between cancers in adolescent and young adults and in older adults
Source: Nat Commun. 2022 Nov 24;13:7223. doi: 10.1038/s41467-022-34959-2 (PMC9700745; doi:10.1038/s41467-022-34959-2)
Supplement: Supplementary file 3 — Description of Additional Supplementary Files [file 41467_2022_34959_MOESM3_ESM.pdf]

Description of Additional Supplementary Files

File Name: Supplementary Data 1
Description: Comparison of AYA and OA clinical characteristics. Statistical differences were assessed using two-sided Fisher's exact test, followed by BenjaminiHochberg method for multiple hypothesis testing.

File Name: Supplementary Data 2
Description: Comparison of TMB between AYAs and OAs. Statistical differences were assessed using two-sided Wilcoxon rank sum test, followed by Benjamini-Hochberg method for multiple hypothesis testing.

File Name: Supplementary Data 3
Description: Comparison of gene mutation rates between AYA and OA.

File Name: Supplementary Data 4
Description: Output of Logistic regression model for gene mutation rate comparison.

File Name: Supplementary Data 5
Description: Comparison of TERT promoter mutations. Statistical differences were assessed using two-sided Fisher's exact test, followed by Benjamini-Hochberg method for multiple hypothesis testing.

File Name: Supplementary Data 6
Description: Comparison of genome instability and gene level copy number alterations. Statistical differences of genome instability were assessed using two-sided Wilcoxon rank sum test, followed by Benjamini-Hochberg method for multiple hypothesis testing.

File Name: Supplementary Data 7
Description: Comparison of gene fusion based on panel MSK468. Statistical differences were assessed using two-sided Fisher's exact test, followed by BenjaminiHochberg method for multiple hypothesis testing.

File Name: Supplementary Data 8
Description: Mutation signatures of hypermutators in AYAs and OAs. Statistical differences were assessed using two-sided Fisher's exact test, followed by Benjamini-Hochberg method for multiple hypothesis testing.

File Name: Supplementary Data 9
Description: Logistic regression model results for gene mutation rate comparison in MSKMET dataset.
