# Supplementary material for: Genomic disparities between cancers in adolescent and young adults and in older adults
Source: Nat Commun. 2022 Nov 24;13:7223. doi: 10.1038/s41467-022-34959-2 (PMC9700745; doi:10.1038/s41467-022-34959-2)
Supplement: Supplementary file 13 — Reporting Summary [file 41467_2022_34959_MOESM13_ESM.pdf]

nature portfolio

Corresponding author(s): Xiaojing Wang

Last updated by author(s): 2022/11/05

Reporting Summary

Nature Portfolio wishes to improve the reproducibility of the work that we publish. This form provides structure for consistency and transparency in reporting. For further information on Nature Portfolio policies, see our Editorial Policies and the Editorial Policy Checklist.

Statistics

For all statistical analyses, confirm that the following items are present in the figure legend, table legend, main text, or Methods section.

n/a	Confirmed	
☐	☒	The exact sample size (*n*) for each experimental group/condition, given as a discrete number and unit of measurement
☐	☒	A statement on whether measurements were taken from distinct samples or whether the same sample was measured repeatedly
☐	☒	The statistical test(s) used AND whether they are one- or two-sided<br>*Only common tests should be described solely by name; describe more complex techniques in the Methods section.*
☐	☒	A description of all covariates tested
☐	☒	A description of any assumptions or corrections, such as tests of normality and adjustment for multiple comparisons
☐	☒	A full description of the statistical parameters including central tendency (e.g. means) or other basic estimates (e.g. regression coefficient) AND variation (e.g. standard deviation) or associated estimates of uncertainty (e.g. confidence intervals)
☐	☒	For null hypothesis testing, the test statistic (e.g. *F*, *t*, *r*) with confidence intervals, effect sizes, degrees of freedom and *P* value noted<br>*Give P values as exact values whenever suitable.*
☒	☐	For Bayesian analysis, information on the choice of priors and Markov chain Monte Carlo settings
☒	☐	For hierarchical and complex designs, identification of the appropriate level for tests and full reporting of outcomes
☐	☒	Estimates of effect sizes (e.g. Cohen's *d*, Pearson's *r*), indicating how they were calculated

Our web collection on statistics for biologists contains articles on many of the points above.

Software and code

Policy information about availability of computer code

Data collection	No software was used for data collection.
Data analysis	All statistical analyses were carried out using R v4.2.0. We used Fisher's exact test to compare clinical and molecular features between AYAs and OAs. Multivariable logistic regression models were constructed in mutation and copy number comparisons. In these models, AYA status was one variable. Other variables included patient sex, histological subtype, and metastatic status. Survival analysis was performed using log-rank test and Kaplan Meier curve was used for visualization. Hazard ratios were determined using Cox proportional hazards model. For mutational signature, we used deconstructSigs to determine the weights of previously reported hypermutation related mutational signatures in COSMIC (version 2, March 2015).

For manuscripts utilizing custom algorithms or software that are central to the research but not yet described in published literature, software must be made available to editors and reviewers. We strongly encourage code deposition in a community repository (e.g. GitHub). See the Nature Portfolio guidelines for submitting code & software for further information.

Data

Policy information about availability of data

All manuscripts must include a data availability statement. This statement should provide the following information, where applicable:

- Accession codes, unique identifiers, or web links for publicly available datasets
- A description of any restrictions on data availability
- For clinical datasets or third party data, please ensure that the statement adheres to our policy

Genomic and clinical data were downloaded from the AACR GENIE project via Synapse (release 9, https://www.synapse.org/#!Synapse:syn7222066/wiki/410924). This study was performed in strict accordance with the recommendations of data access guideline of AACR project GENIE datasets. TCGA genomic data (mc3), molecular subtype data and clinical data were downloaded from PanCanAtlas (https://gdc.cancer.gov/about-data/publications/). MSK-IMPACT immune checkpoint inhibitor therapy cohort was downloaded from https://www.nature.com/articles/s41588-018-0312-8#Sec7. The processed data generated in this study are provided in the Supplementary Information/Source Data file. The remaining data are available within the Article, Supplementary Information or Source Data file Source data are provided with this paper.

Human research participants

Policy information about studies involving human research participants and Sex and Gender in Research.

Reporting on sex and gender	We used self-reported sex information in GENIE data. For mutation/CNV rate comparison between AYAs and OAs, we used multivariable logistic regression analysis with patient sex as one of the variables.
Population characteristics	Not applicable.
Recruitment	Not applicable.
Ethics oversight	Not applicable.

Note that full information on the approval of the study protocol must also be provided in the manuscript.

Field-specific reporting

Please select the one below that is the best fit for your research. If you are not sure, read the appropriate sections before making your selection.

☒ Life sciences ☐ Behavioural & social sciences ☐ Ecological, evolutionary & environmental sciences

For a reference copy of the document with all sections, see nature.com/documents/nr-reporting-summary-flat.pdf

Life sciences study design

All studies must disclose on these points even when the disclosure is negative.

Sample size	Sample sizes were not predetermined as we aimed to use all samples in GENIE data with available mutation, CNVs and fusion data.
Data exclusions	We used 'AGE_AT_SEQ_REPORT' to determine AYA (15-39 years old) and OA groups (>39 years old), assuming the time of clinical sequencing is close to age at the time of diagnosis because of the fast turnaround for clinical sequencing. We excluded cases without precise age information (including '<18', n=3785, and 'Unknown' n=6215) from our study. Clinical and epidemiological characteristics of cancers in AYAs was examined in 19 cancer types that had more than 100 cases in both AYAs and OAs.
Replication	This is a population based study, so the replication is not relevant. But we confirmed our findings in another independent cohort (TCGA).
Randomization	We didn't apply any randomization method in our study. Instead, we built a logistic regression model to control possible confounding factors or characteristics when we compared the gene alteration difference between AYAs and OAs.
Blinding	This is a population based study to compare genomic differences between AYA and OA groups. The two groups were divided based on patient age, so blinding is not applicable.

Reporting for specific materials, systems and methods

We require information from authors about some types of materials, experimental systems and methods used in many studies. Here, indicate whether each material, system or method listed is relevant to your study. If you are not sure if a list item applies to your research, read the appropriate section before selecting a response.

Materials & experimental systems

n/a	Involved in the study
[x]	[ ] Antibodies
[x]	[ ] Eukaryotic cell lines
[x]	[ ] Palaeontology and archaeology
[x]	[ ] Animals and other organisms
[x]	[ ] Clinical data
[x]	[ ] Dual use research of concern

Methods

n/a	Involved in the study
[x]	[ ] ChIP-seq
[x]	[ ] Flow cytometry
[x]	[ ] MRI-based neuroimaging
